# Elasto-Plastic Fracture Mechanics Analysis of the Effect of Shot Peening on 300M Steel

**DOI:** 10.3390/ma14133538

**Published:** 2021-06-25

**Authors:** Shuai Hou, Zhihai Cai, Youli Zhu, Qizhi Zhao, Yong Chen, Han Gao, Hongbo Wang, Jing Li

**Affiliations:** 1 State Key Laboratory of NBC Protection for Civilian, Institute of Chemical Defense, Academy of Military Sciences, Zhijiang 443200, China; houshuai2012@sina.com (S.H.); zhaoqz1214@163.com (Q.Z.); chenyong_152@sina.com (Y.C.); gh8562@126.com (H.G.); wanghongbo1100@sina.com (H.W.); 2 National Engineering Research Center for Remanufacturing, Army Academy of Armored Forces, Beijing 100072, China; gzulijing@163.com

**Keywords:** shot peening (SP), residual stress, J-integral, semi-elliptic surface crack, crack propagation rate, crack kinking angle

## Abstract

A modified J-integral calculation method is adopted to fix the problem of the quantitative evaluation of the crack propagation of shot-peened structures. Considering the residual stress, residual strain, and residual strain energy, the effect of shot peening on the J-integral parameters of semi-elliptic surface crack fronts is quantitatively calculated and a method is provided for the performance evaluation of the shot peening layer. First, the shot peening process is simulated, then the fatigue crack is generated by changing the constraint condition and a far-field load is applied to calculate the J-integral parameters, crack propagation rate, and crack kinking angle. The effects of different crack depths and shot velocities on the fracture parameters are analyzed. The results show that the reduction in the J-integral value after shot peening decreases with the increase in the crack depth when the shot velocity is a certain value, which indicates that shot peening is more beneficial for suppressing the fatigue crack propagation. When the crack depth is greater than the depth of the compressive stress layer, shot peening accelerates the crack propagation. The reduction in the J-integral value decreases with the increase in shot velocity when the crack depth is a certain value; therefore, increasing shot velocity is more beneficial for retarding fatigue crack propagation.

## 1. Introduction

Shot peening is a strengthening process that uses a large number of shots to impact surface materials to cause circulating plastic deformation [1,2]. It is used to improve the fatigue properties of materials by creating residual compressive stresses in the surface layer of the material while improving the microstructure, and it is used in a wide range of mechanical applications, such as in the aerospace and automotive industries [3,4,5].

As the requirements for mobility and fatigue reliability continue to increase, the application of shot peening is receiving more and more attention. In addition to the continuous improvement of shot peening processes and equipment, the assessment of the life of shot-peened components has also become a hot topic of research, but currently the assessment of the life of shot-peened structures relies mainly on fatigue testing [6,7]. This is not only time-consuming and costly but also has a large dispersion of test results. There is a lack of effective methods for quantitatively assessing the effect of shot peening on fatigue crack propagation life, as assessing crack extension life requires the calculation or measurement of fracture mechanical parameters, such as the J-integral or stress intensity factor at the crack front. Due to the shallowness of the shot peening layer (a residual compressive stress layer less of than 1 mm and a microstructure strengthening layer of less than 0.2 mm), it is difficult to prepare standard CT(Compact Tension) specimens and, therefore, difficult to measure the J-integral of the shot peening layer using experimental methods, which is currently one of the main difficulties when considering the role of the shot peening layer for propagation life in the design phase of structural life.

The use of fracture mechanics methods to calculate the effect of shot peening on the fracture parameters of the crack front is a viable approach. The method currently used is mainly based on the theory of linear elastic fracture mechanics, where the residual stress is linearly superimposed on the stress intensity factor of the external load acting alone [8,9]. Gao [10] used the XRD method to obtain the distribution curve of shot peening residual stresses along the depth, then used the weight function method to calculate the stress intensity factor of a single-sided notched tensile specimen. Lv [11] used Robertson’s formula [12] to fit the shot peening residual stress curve to establish a two-dimensional four-point bending finite element model, calculate the crack tip stress intensity factor, and consider the crack closure effect. Liu [13] developed a two-dimensional cohesive finite element model, fitted the shot peening residual stresses from the literature, and calculated the crack tip stress intensity factor under cyclic loading.

The above methods only fit the residual stress distribution after shot peening, based on linear elastic fracture mechanic parameters (stress intensity factors) that do not take into account the effects of work hardening (plastic deformation) and residual strain energy formed by shot peening and reduce it to a two-dimensional problem, ignoring the complexity of shot peening residual stresses, which inevitably leads to errors in the calculation of fracture parameters at the crack front. Currently, the main analytical method is based on the theory of linear elastic fracture mechanics [14,15], where the residual stress is linearly superimposed on the external load to solve for the stress intensity factor. The weight function method, the cohesion model, and the finite element method are essentially linear elastic fracture mechanics, only differing in their numerical methods. Shot peening is a process that causes the plastic deformation of the material, and the linear elastic fracture mechanics parameters do not assess the strength of the stress field at the crack front in the plastic zone, so this approach actually oversimplifies the problem. Meanwhile, in the presence of shot peening residual stress, residual strain, and residual strain energy, the conventional J-integral calculation is no longer path-independent and, therefore, cannot be used as a fracture parameter. Lei [16] presents a modified J-integral formulation without accounting for body forces and crack face forces and demonstrates its path independence, in which parameters such as residual stress, residual strain, and residual strain energy are taken into account, providing a theoretical basis for the quantitative calculation of crack propagation life in shot-peened structures.

A three-dimensional finite element modelling simulation of the shot peening process was carried out and fatigue cracks were generated by varying the constraints and applying far-field loads. A modified J-integral was then used to calculate the J-integral values, crack expansion rates, and crack kinking angles for the semi-elliptical surface cracks. The effects of the shot peening process and crack depth on the above fracture parameters were investigated to provide a computational method for quantitatively assessing the fatigue crack propagation life of shot-peened layers.

## 2. Calculation of the Modified J-integral, Crack Propagation Rate, and Crack Kinking Angle

### 2.1. Modified J-Integral

The stress intensity factor and energy release rate derived on the basis of linear elastic fracture mechanics are only applicable to small-range yielding problems. To solve fracture problems with large-range yielding, elasto-plastic fracture mechanics were developed. The J-integral is a fracture parameter which is an extension of the energy release rate principle. Rice [17] proposed and proved the path-independent property of the J-integral.

(1) $$J=\underset{\Gamma \to 0}{\lim}\int_{\Gamma }\left(W\delta_{1i}-\sigma_{ij}\frac{\partial u_{j}}{\partial x_{1}}\right)n_{i}ds$$

where *W* is the strain energy density of the cracked body, *σ_ij_* is the stress tensor acting on the arc element d*s*, *u_j_* is the displacement vector at the point on d*s*, and *δ_ij_* is the Kronecker notation. *n_i_* is the directional cosine of the outer normal of d*s*. Γ is the curve around the crack tip, starting at the lower surface of the crack and ending at the upper surface of the crack in a counterclockwise direction (positive direction of d*s*).

The J-integral of Equation (1) is independent of the path under purely mechanical loading, without regard to physical forces and crack face forces, and without regard to residual stresses. When residual stresses are present, as in the case of welded structural cracks or hole cold expansion, the J-integral values calculated using Equation (1) show a greater path dependence [18,19]. Lei [16] proposes to treat the residual stress problem as an initial strain problem and to modify Equation (1) to ensure the path-independence of the J-integral. The total strain $\varepsilon_{ij}$ can be decomposed into two components: the total mechanical strain $\varepsilon_{ij}^{m}$ and the initial strain $\varepsilon_{ij}^{0}$.

(2) $$\varepsilon_{ij}=\varepsilon_{ij}^{m}+\varepsilon_{ij}^{0}$$

The mechanical strains $\varepsilon_{ij}^{m}$ and stresses $\sigma_{ij}$ satisfy the material constitutive equations. Without accounting for physical forces and crack surface forces, Equation (1) is modified to:

(3) $$J=\underset{\Gamma \to 0}{\lim}\int_{\Gamma }^{}(W^{m}\delta_{1i}-\sigma_{ij}\frac{\partial u_{j}}{\partial x_{1}})n_{i}ds+\int_{A}^{}\sigma_{ij}\frac{\partial \varepsilon_{ij}^{0}}{\partial X_{1}}dA$$

where *W*^m^ is the mechanical strain energy density:

(4) $$W^{m}=\int_{A}^{\varepsilon_{}^{m}}\sigma_{ij}d\varepsilon_{ij}^{m}$$

*A* is the closed-loop surface enclosed by the path Γ, and as *A* tends to 0 it tends toward the crack tip. The plastic strain ${\left.\varepsilon_{ij}^{p}\right|}_{\mathrm{ini}}$ in the initial state is used as the initial strain $\varepsilon_{ij}^{0}$ in the subsequent analysis:

(5) $$\varepsilon_{ij}^{0}={\left.\varepsilon_{ij}^{p}\right|}_{\mathrm{ini}}$$

The elastic strains, including the initial elastic strain, are all recoverable and, therefore, all contribute to the mechanical strain; therefore, the mechanical strain $\varepsilon_{ij}^{m}$ is defined as:

(6) $$\varepsilon_{ij}^{m}=(\varepsilon_{ij}^{e}+\varepsilon_{ij}^{p})-{\left.\varepsilon_{ij}^{p}\right|}_{\mathrm{ini}}$$

where $\varepsilon_{ij}^{e}$ is the total elastic strain and $\varepsilon_{ij}^{p}$ is the total plastic strain.

From Equations (2) and (6), the general form of the initial strain can be obtained from the total strain in the initial state $\varepsilon_{ij}$ and the total elastic mechanical strain $\varepsilon_{ij}^{e}$:

(7) $$\varepsilon_{ij}^{0}={\left(\varepsilon_{ij}-\varepsilon_{ij}^{e}\right)}_{\mathrm{ini}}$$

Since the total plastic strain $\varepsilon_{ij}^{p}$ includes the plastic strain in the initial state ${\left.\varepsilon_{ij}^{p}\right|}_{\mathrm{ini}}$, the strain energy density *W* in Equation (3) should be:

(8) $$W=W^{t}-{\left.W^{p}\right|}_{\mathrm{ini}}$$

where *W*^t^ is the total strain energy density and ${\left.W^{p}\right|}_{\mathrm{ini}}$ is the plastic work density in the initial state.

### 2.2. Crack Propagation Rate

Under plane strain conditions, the stress intensity factor *K* and the *J* integral are related as follows:

(9) $$K=\sqrt{\frac{JE}{1-\upsilon^{2}}}$$

where *E* is the modulus of elasticity and *υ* is the Poisson’s ratio. From the Paris formula [20], the crack propagation rate can be obtained as:

(10) $$da/dN=C{\left(\Delta K\right)}^{m}$$

Crack propagation condition is:

(11) $$\Delta K>\Delta K_{\mathrm{th}}$$

where Δ*K* is the stress intensity factor range for *R* > 0, Δ*K* = *K*_max_ − *K*_min_ and for *R* < 0, Δ*K* = *K*_max_. Δ*K*_th_ is the threshold stress intensity factor range.

### 2.3. Crack Kinking Angle

The main crack extension direction criteria are the maximum tangential stress (MTS), minimum strain energy density (MSED), maximum stress intensity factor (MSIF), maximum energy release rate (MERR), local symmetry (LS), and crack tip opening displacement (CTOD). The MTS criterion is an explicit algorithm which is simpler and less computationally intensive than the MERR criterion. It uses an implicit algorithm and is more commonly used in practical problems. Research has shown that there is very little difference in the calculation of these two criteria [21], and in this paper, the MTS criterion is used for the calculation.

**Figure 1:**
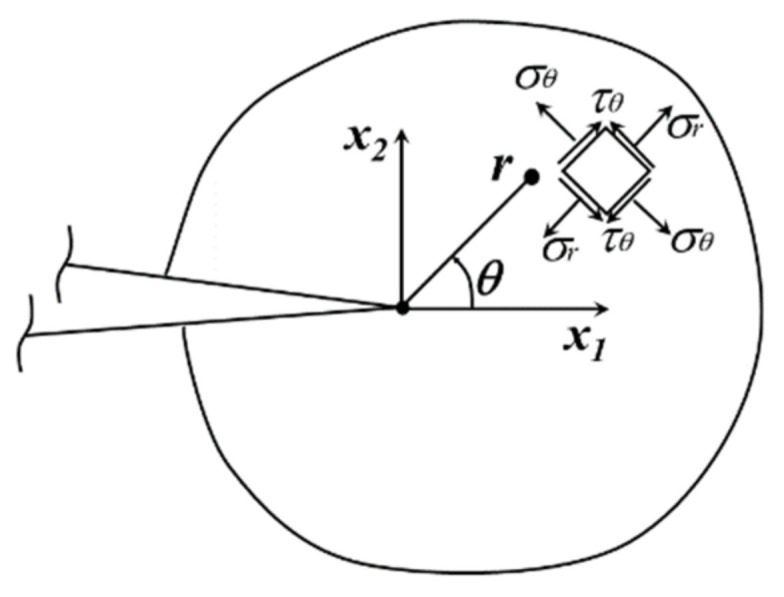
Schematic diagram of the MTS criterion for the crack kinking angle.

The MTS criterion assumes that the crack will expand in a direction perpendicular to the maximum circumferential stress (*σ_θθ_*) in the polar coordinate system of the crack tip (as shown in Figure 1). In the crack tip polar coordinate system (*r*,*θ*), the circumferential stresses (*σ_θθ_*) for type I and type II composite cracks are given by [22]:

(12) $$\sigma_{\theta \theta }=\frac{1}{2\sqrt{2\pi r}}\cos\left(\frac{\theta }{2}\right)\left[K_{I}\cos^{2}(\theta )-3K_{\mathrm{II}}\sin(\theta )\right]$$

The crack kinking angle *θ* is:

(13) $$\theta =\arccos\frac{3K_{\mathrm{II}}{}^{2}+\sqrt{K_{I}{}^{4}+8K_{I}{}^{2}K_{\mathrm{II}}{}^{2}}}{K_{I}{}^{2}+9K_{\mathrm{II}}{}^{2}}$$

where *θ* is negative for *K*_II_ > 0 and positive for *K*_II_ < 0.

## 3. Finite Element Model of Shot Peening with Three-Dimensional Semi-Elliptical Surface Cracks

The length, width, and thickness of the target were *x* = 10 mm, *y* = 6 mm, and *z* = 3 mm, respectively, and a semi-elliptical surface crack was created in the symmetry plane (*Y* = 3 mm). The crack half-length was *c* = 1 mm, and six depths of cracks—0.3 mm, 0.5 mm, 0.8 mm, 1.0 mm, 1.2 mm, and 1.5 mm—were created (the maximum depth is noted as *a*).

The material parameters of 300M steel (40CrNi2Si2MoVA) [23] were modulus of elasticity *E* = 200 GPa, Poisson’s ratio *υ* = 0.3, and ρ = 7800 Kg/m^3^. A bilinear kinematic hardening model was used. The yield strength was 1614 MPa and the hardening modulus was 2885 MPa.

The fatigue crack propagation parameters for 300M steel [24] were *C* = 3.847 × 10^−8^, *m* = 2.55. The threshold stress intensity factor range [25] was Δ*K*_th_ = 2.9 $\mathrm{MPa}\sqrt{m}$. The crack propagation rate was calculated for fatigue loading with a stress ratio *R* = 0 and a maximum stress *σ_max_* = 1100 MPa.

**Figure 2:**
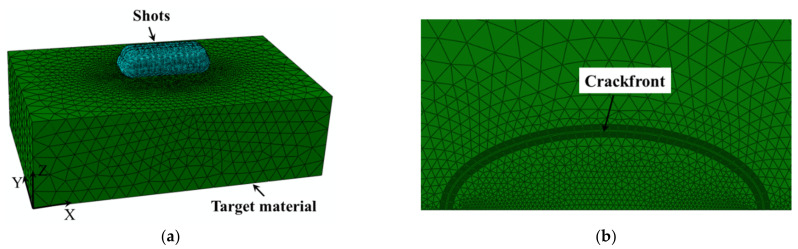
Shot peening finite element model and crack front meshing. (**a**) Shot peening FEM model meshing. (**b**) Crack surface mesh refinement.

The shot was set up as rigid (divided by R3D4 element), with a diameter of 0.8 mm, a density of 7800 Kg/m^3^, and an initial velocity of 40 m/s. In order to cover the cracked area uniformly, 75 shots were used in the model; see Figure 2a. The surface coverage was approximately 100%. The tangential friction factor between the shot and the target surface was 0.2 [26], and the interaction between the crack surfaces (after cracking) was set to hard contact [27]. The contact state was tracked using the small sliding method, and the contact algorithm used the penalty function method.

A wedge-shaped singular element containing 1/4 nodes (C3D6) was used for the first ring of the crack front; a linear hexahedral element (C3D8) was used for the second to seventh rings of the crack front, and a tetrahedral element (C3D4) was used for the rest.

Due to the large stress-strain gradient in the shot peening region, mesh refinement was carried out to ensure calculation accuracy, with a minimum mesh size of approximately 0.005 mm, as Figure 2b shows. The calculation process was as follows:

(1) An explicit dynamics algorithm was used to simulate the shot peening process. All degrees of freedom of the nodes on the bottom surface *Z* = 0 were constrained, a binding constraint was used to constrain the two crack surface node degrees of freedom, and the initial velocity of the shot reference point was set. In order to reduce the amount of calculations, the arrangement of the shots was designed in 2 layers of 3 rows each, with a 0.2 mm interval between adjacent shots within the layer and layer height of 0.2 mm (in the Z direction), with the first layer deviating by 0.1 mm in the X direction for a total of 75 shots, achieving 100% coverage. The shots struck the surface of the specimen at a set velocity layer by layer.
(2) When simulating the unloading process, the model was copied, the shots were removed, and the remaining boundary conditions were left unchanged. The predefined fields of ABAQUS FEM(Finite Element Method) Software [28] were used to import the stresses and strains calculated in the previous step, and the analysis step was changed to static analysis. An implicit algorithm was used to calculate the equilibrium state after shot peening.
(3) In crack generation, constraints were removed from the nodes on the bottom surface *Z* = 0, all degrees of freedom of the nodes were constrained on the *Y* = 0 surface, and the binding constraints were removed from the nodes on the crack surface. The equilibrium state was calculated and used as the initial state.
(4) A far-field tensile load was applied. Tensile load (1100 MPa) was applied to the surface node at *Y* = 6, and the crack front modified J-integral was calculated using Equation (3). For a comparative analysis, the J-integral values of the crack fronts without shot peening were calculated using Equation (1) for the same semi-elliptical surface cracks and tensile loads.

## 4. Results and Discussion

### 4.1. Stress and Strain State after Shot Peening and Cracking

**Figure 3:**
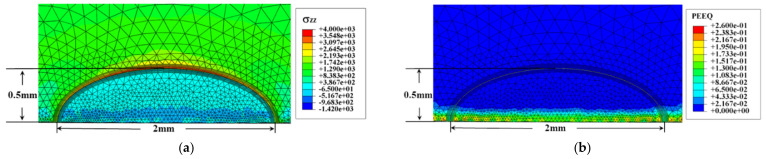

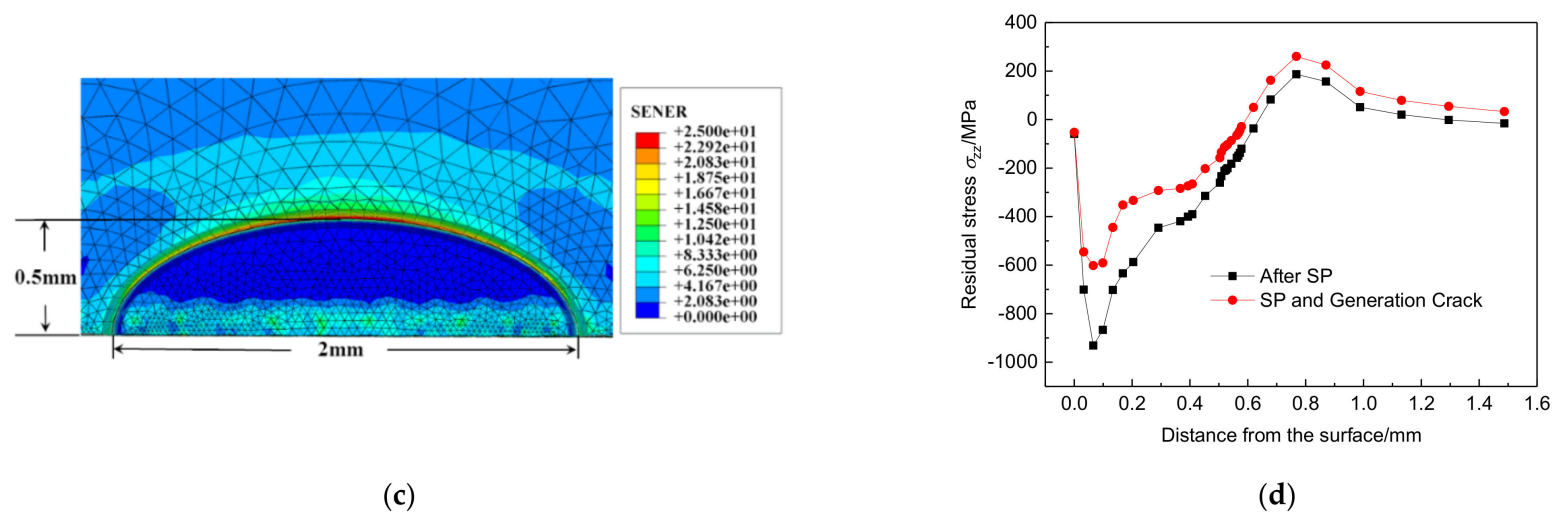
Parameters on the crack face after shot peening and the generation of a 0.5 mm-deep crack. (**a**) Residual stresses perpendicular to the crack plane. (**b**) Equivalent plastic strain. (**c**) Elastic strain energy density. (**d**) Distribution of residual stresses *σ_zz_* in the symmetry axis on the symmetry plane of the crack.

As an example, the residual stress, residual strain, and residual elastic strain energy density on the crack surface were obtained by shot peening, unloading, and generating a 0.5 mm deep crack, as shown in Figure 3. The residual stresses exhibited a large singularity at the crack tip (Figure 3a), and the residual stress *σ_zz_* perpendicular to the crack face on the symmetry axis was extracted, neglecting the crack tip singularity. The distribution curve is shown in Figure 3d. It can be seen that the residual stress was released and the depth of the residual compressive stress *σ_zz_* was about 0.5 mm; beyond 0.5 mm, it turns into residual tensile stress, and the residual tensile stress was at its maximum at about 0.8 mm from the surface. A plastic deformation layer (work-hardened layer, as shown in Figure 3b) of approximately 0.2 mm depth was produced after shot peening. The elastic strain energy density layer was approximately 0.22 mm deep (Figure 3c). As can be seen from Equation (3), shot peening produces changes in the material state (residual stress, residual strain, and residual elastic strain energy) that will affect the crack front fracture parameters, which are not addressed by models of linear elastic fracture mechanics.

**Figure 4:**
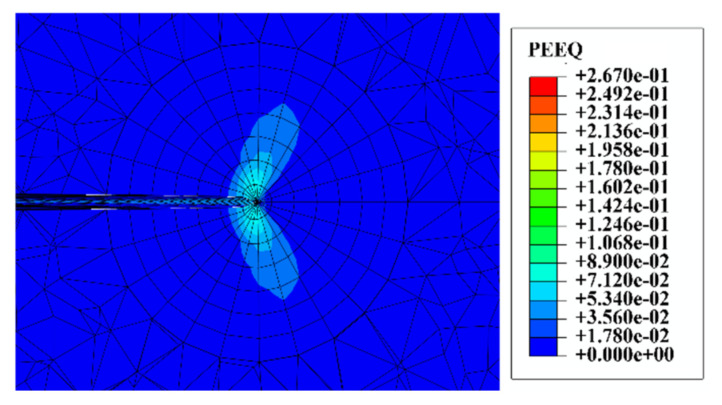
Equivalent plastic strain at the deepest point of the crack.

As stress singularities inevitably arise at the crack tip, J-integral values close to the crack front are less accurate and J-integrals converging to the crack front are not physically meaningful [29]. In addition, under small yield conditions the integration domain of the J-integral should bypass the plastic zone of the crack tip as far as possible to improve the accuracy of the numerical calculation [30]. Figure 4 shows the equivalent plastic strain contour map at the deepest point of the crack. Ring 5 encloses the entire plastic zone, so any of rings 5 to 7 can be chosen as the J-integral value.

### 4.2. The Effect of Shot Peening on Cracks of Different Depths

#### 4.2.1. Effect of Shot Peening on J-Integral

**Figure 5:**
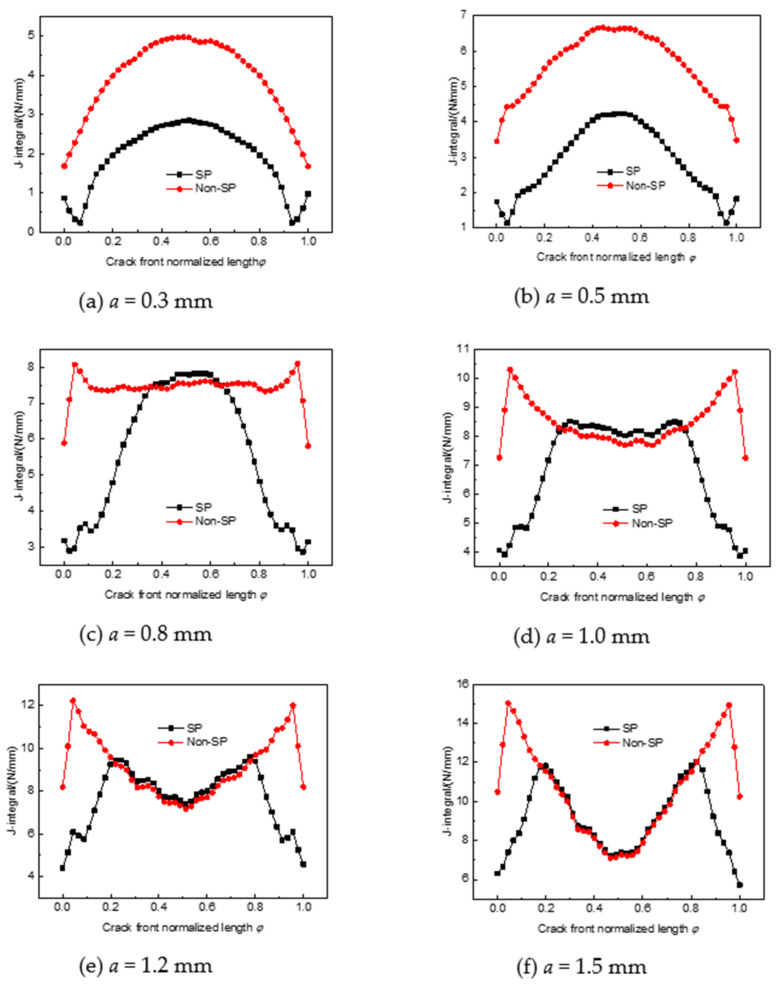
J-integration of crack fronts for peened and non-shot-peened parts. (**a**) a = 0.3 mm; (**b**) a = 0.5 mm; (**c**) a = 0.8 mm; (**d**) a = 1.0 mm; (**e**) a = 1.2 mm; (**f**) a = 1.5 mm.

For comparison purposes, the horizontal coordinate is the normalized crack length φ = Δ*l*/*L*; Δ*l* is the length of a point on the crack along the crack front curve to the surface endpoint, and *L* is the total length of a semi-elliptical surface crack. Figure 5 shows the J-integral of the crack fronts at different depths without shot peening and after shot peening. When the crack is shallow, the maximum value of J-integral is located at the deepest point of the crack, and the distribution curve of J-integral values after shot peening is lower than that of a non-shot-peened structure. When the crack depth is 0.3 mm, the J-integral value at the deepest point of the crack decreases from 4.97 N/mm to 2.83 N/mm, a reduction of approximately 43%, and when the crack depth is 0.5 mm, the reduction is approximately 36%, which indicates that the reduction in the J-integral value after shot peening decreases with increasing crack depth when the crack is within the strengthening layer. Therefore, shot peening is more beneficial for inhibiting the expansion of fatigue shallow cracks. When the crack depth was greater than 0.8 mm, the J-integral value at the deepest point of the crack after shot peening exceeded that of the non-shot-peened parts, indicating that shot peening did not inhibit the expansion of the fatigue crack in the residual tensile stress zone. The distribution of the J-integral was M-shaped, indicating that the fatigue hazard point shifted to the ends of the crack.

#### 4.2.2. Effect on the Crack Propagation Rate

**Figure 6:**
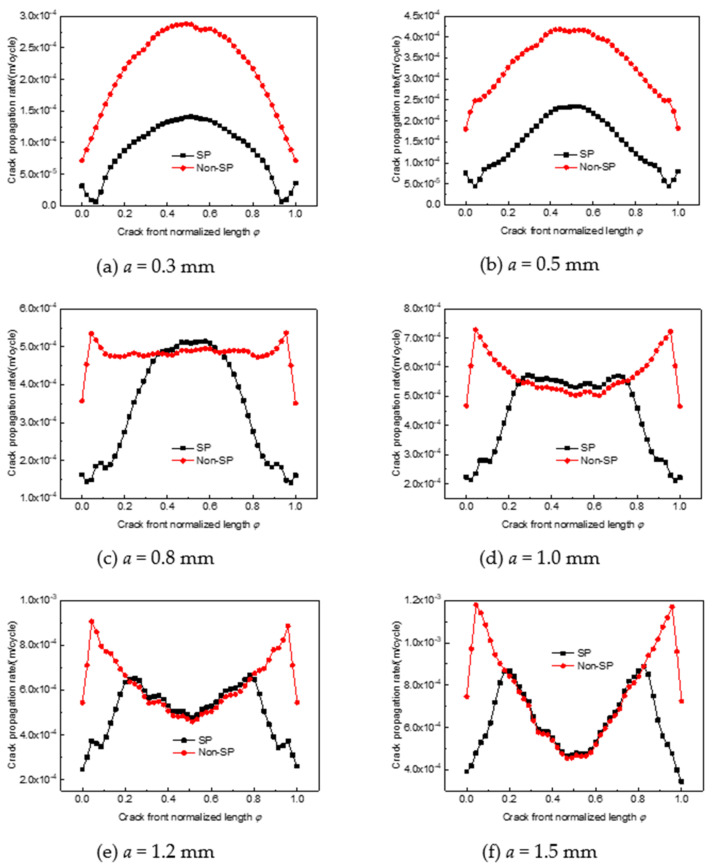
Crack front extension rate for peened and non-shot-peened parts. (**a**) a = 0.3 mm; (**b**) a = 0.5 mm; (**c**) a = 0.8 mm; (**d**) a = 1.0 mm; (**e**) a = 1.2 mm; (**f**) a = 1.5 mm.

The crack propagation rate at the crack front is shown in Figure 6. It can be seen that the crack propagation rate is significantly reduced after shot peening when the crack depth is shallow, especially because when the crack depth is 0.3 mm, the maximum crack propagation rate under a given load is reduced by approximately 50%. As the crack depth increases, the effect of shot peening to inhibit crack expansion decreases. At a crack depth of 0.8 mm or 1.0 mm, the crack propagation rate at the deepest point after shot peening exceeds that of the non-shot-peened structure, indicating that residual tensile stresses increase the rate of crack expansion. The crack propagation rate at both ends of the crack is still lower than that of the non-shot-peened structure as it is still within the shot peening layer, which further suggests that shot peening only inhibits the expansion of the crack within the peening layer.

#### 4.2.3. Effect on Crack Kinking Angle

**Figure 7:**
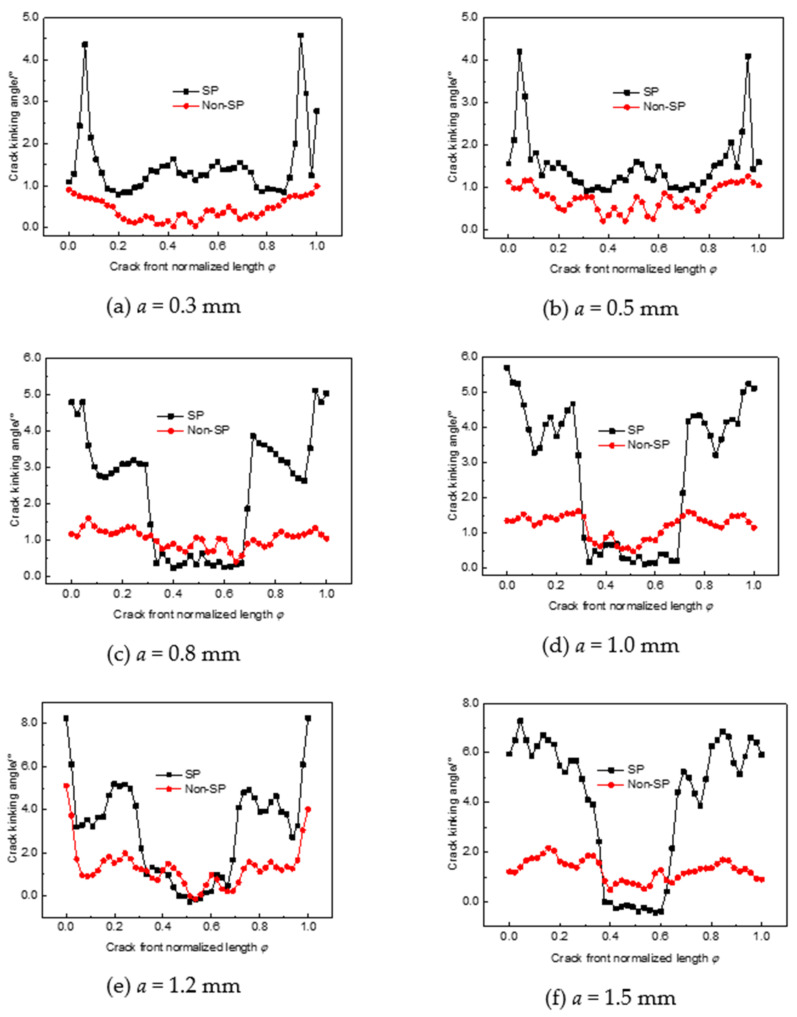
Crack kinking angle of peened and non-shot-peened parts. (**a**) a = 0.3 mm; (**b**) a = 0.5 mm; (**c**) a = 0.8 mm; (**d**) a = 1.0 mm; (**e**) a = 1.2 mm; (**f**) a = 1.5 mm.

The crack kinking angles after shot peening and without shot peening are shown in Figure 7. Compared to non-shot-peened parts, the crack kinking angle of different depths after shot peening is relatively large, especially for shallow cracks with a crack depth of less than 0.5 mm, and the crack front has an overall larger kinking angle. This is due to the uneven stress, strain, and strain energy produced by shot peening, which deflects the crack expansion direction. This means that cracks in shot-peened structures will have a more tortuous path of expansion in the early stages of expansion and a longer crack expansion life. As the crack depth increases, the kinking angle within the peening layer increases, while the kinking angle of the part of the crack front beyond the peening layer depth is close to that of the non-shot-peened part, which further indicates that shot peening only inhibits crack expansion within the peening layer.

### 4.3. Effect of Shot Velocity on Crack Extension

#### 4.3.1. Effect on J-Integral

**Figure 8:**
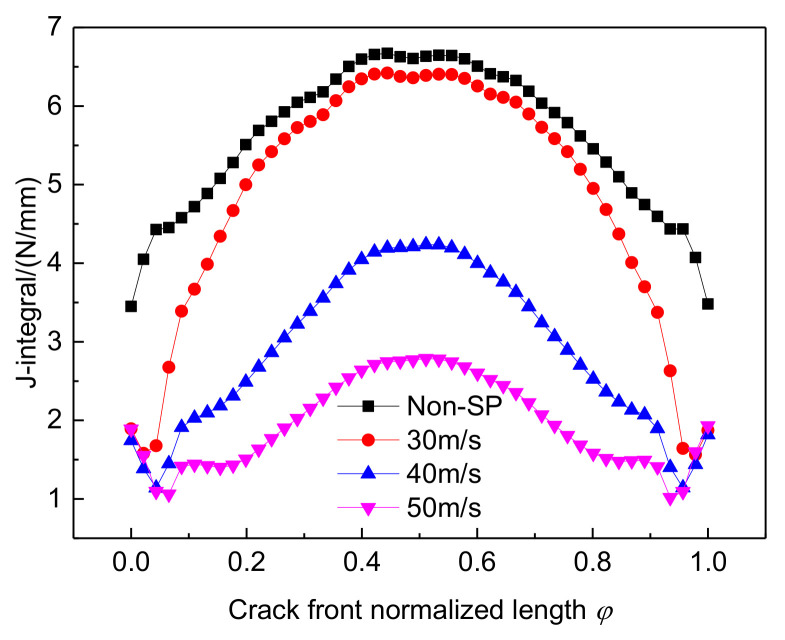
J-integral of crack fronts at different shot velocities.

To investigate the effect of shot velocity, shot peening was carried out using initial velocities of 30 m/s, 40 m/s, and 50 m/s, respectively, changing the constraint to introduce a 0.5 mm-deep crack and adding a far-field tensile load, with other conditions being the same. The J-integral of the crack front obtained from Equation (3) is shown in Figure 8. The J-integral curve after shot peening is reduced to below that of non-shot-peened parts. Comparing the J-integral values of the deepest point of the crack at different shot velocities, the difference between the J-integral values of the shot-peened and the non-shot-peened was relatively small when the shot velocity was 30 m/s. The J-integral value decreases significantly as the shot speed increases, with a 58% decrease in the J-integral value at the crack front at an initial shot speed of 50 m/s. This indicates that a higher shot velocity is more beneficial for inhibiting crack expansion.

#### 4.3.2. Effect on Crack Tip Opening Displacement

**Figure 9:**
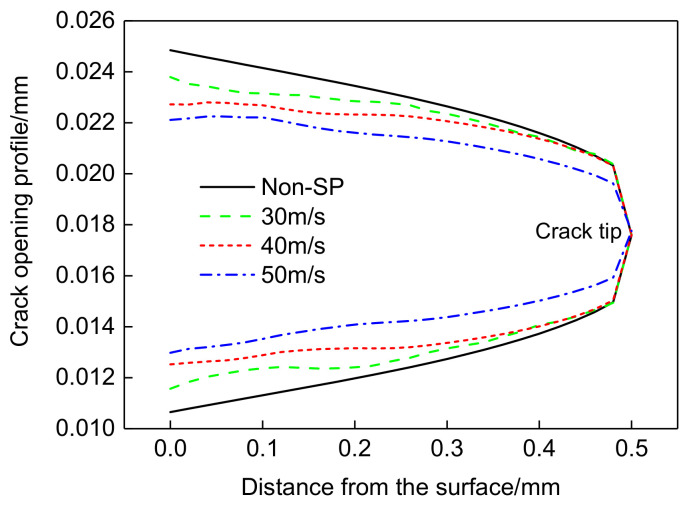
Crack opening profile under far-field loading at different shot velocities.

The nodal coordinates of the upper and lower crack surface symmetry lines were extracted to obtain the crack surface profile curve, as shown in Figure 9, from which the crack tip opening displacement can be directly calculated and observed. At a shot velocity of 30 m/s, there is a small decrease in crack tip opening displacement compared to non-shot-peened parts, and as the shot velocity increases the crack tip opening displacement decreases significantly, directly demonstrating the inhibitory effect of shot peening on crack expansion.

#### 4.3.3. Effect on the Crack Propagation Rate

**Figure 10:**
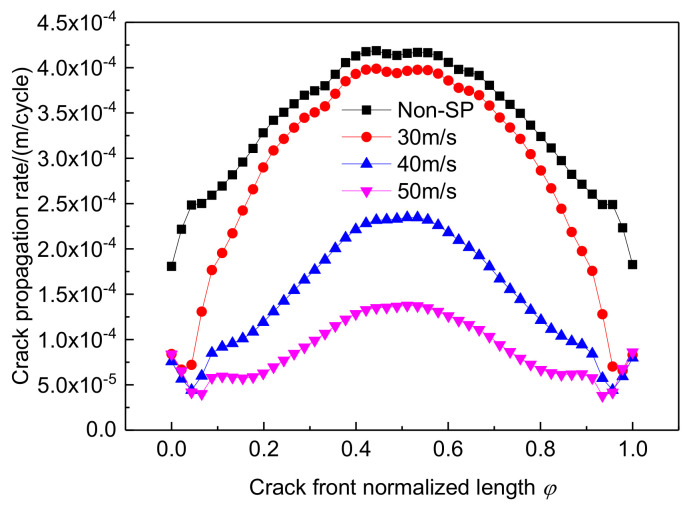
Crack propagation rate after shot peening at different shot velocities.

Figure 10 shows the results for the crack propagation rate at the deepest point of the crack. Compared to non-shot-peened parts, the crack propagation rate is significantly lower after shot peening, with an overall decrease in the propagation rate curve. At a shot speed of 30 m/s, the crack propagation rate at the deepest point decreases to a lesser extent, and as the shot speed increased, the rate of crack expansion decreased significantly, especially because when the shot speed reached 50 m/s, the propagation rate at the deepest point of the crack decreased by 67%. This indicates that when the crack depth is certain, increasing the shot speed is more beneficial for inhibiting the crack expansion.

## 5. Conclusions

The modified J-integral evaluates the effect of residual stress, equivalent plastic strain, and strain energy density on cracking and can be used to evaluate the crack front stress field strength of shot-peened parts under far-field stresses. Methods are proposed to assess the residual strength and crack propagation life of shot-peened structures. The main conclusions are as follows:

A method is proposed to quantitatively assess the effect of the shot peening process on fatigue cracking using the theory of elastic-plastic fracture mechanics. First, the shot peening process is calculated, then the crack is initiated and the fracture parameters at the crack front are calculated using a modified J-integral. Finally, the crack expansion rate and expansion angle are calculated.

The J-integral value decreases with increasing crack depth for a given shot velocity. Shot peening is beneficial for inhibiting the expansion of shallow fatigue cracks. When the crack depth exceeds the residual compressive stress depth and enters the residual tensile stress zone, shot peening will accelerate the crack expansion.

The crack expansion angle is relatively large after shot peening and the crack expansion path is deflected, which helps to extend the fatigue crack expansion life.

When the crack depth is certain, the larger shot velocity is beneficial for suppressing the crack expansion and the J-integral value, crack tip opening displacement, and crack propagation rate are all reduced. The maximum J-integral value of a 0.5 mm-deep semi-elliptical surface crack is reduced by approximately 58%, and the maximum propagation rate is reduced by approximately 67% at a shot velocity of 50 m/s.

## Data Availability

The data presented in this study are available on request from the corresponding author.
